# MicroRNA-125b Induces Metastasis by Targeting STARD13 in MCF-7 and MDA-MB-231 Breast Cancer Cells

**DOI:** 10.1371/journal.pone.0035435

**Published:** 2012-05-31

**Authors:** Feng Tang, Rui Zhang, Yunmian He, Meijuan Zou, Le Guo, Tao Xi

**Affiliations:** 1 School of Life Science and Technology, China Pharmaceutical University, Nanjing, People’s Republic of China; 2 Jiangsu Key Laboratory of Carcinogenesis and Intervention, China Pharmaceutical University, Nanjing, People’s Republic of China; University of Illinois at Chicago, United States of America

## Abstract

MicroRNAs (miRNAs) are a class of small noncoding RNAs that regulate gene expression by targeting mRNAs to trigger either translation repression or mRNA degradation. miR-125b is down-regulated in human breast cancer cells compared with the normal ones except highly metastatic tumor cells MDA-MB-231. However, few functional studies were designed to investigate metastatic potential of miR-125b. In this study, the effects of miR-125b on metastasis in human breast cancer cells were studied, and the targets of miR-125b were also explored. Transwell migration assay, cell wound healing assay, adhesion assay and nude mice model of metastasis were utilized to investigate the effects of miR-125b on metastasis potential *in vitro* and *in vivo*. In addition, it was implied STARD13 (DLC2) was a direct target of miR-125b by Target-Scan analysis, luciferase reporter assay and western blot. Furthermore, activation of STARD13 was identified responsible for metastasis induced by miR-125b through a siRNA targeting STARD13. qRT-PCR, immunofluorescent assay and western blot was used to observe the variation of Vimentin and α-SMA in breast cancer cells. In summary, our study provided new insights into the function of miR-125b during the metastasis of breat cancer cells and also suggested the role of miR-125b in pro-metastasis by targeting STARD13.

## Introduction

MicroRNAs (miRNAs), a series of endogenous, non-coding and single-strand RNA approximately containing 22 nucleotides, regulate messenger RNAs at the post-transcriptional level through binding to complementary sequences in un-translated regions (UTR) of target mRNAs bearing fully complementary target sites, inducing their degradation or repressing translation. [1], [2] miRNAs target abundant genes which regulate a great variety of biological effects, including proliferation, apoptosis, stem cell self-renewal and differentiation. [3], [4] Moreover, expression of miRNA is significantly modulated in a tissue- and developmental stage-specific manner. [5] Recent studies analyzed miRNA profiles and functions in cancer provided valuable information on the molecular pathogenesis of several tumor types, including breast, lung, colon and prostate cancer, hepatocellular carcinoma and glioblastoma [6], [7], [8]. It is widely accepted that miRNAs play an important role in all steps of tumorigenesis.

miR-125b, a human homologue of lin-4, downregulated in bladder, ovarian and breast tumors with functions as a tumor suppressor. [9], [10], [11], [12] However, miR-125b not only over-expresses in pancreatic cancer, oligodendroglial tumors, prostate cancer, myelodysplastic syndromes and acute myeloid leukemia, but also promotes cell proliferation in prostate cancer cells, enhances invasive potential in urothelial carcinomas and suppresses p53-dependent apoptosis in human neuroblastoma cells. [13], [14], [15], [16] Latest report identified miR-125b as a basal-like microRNAs and was significantly elevated in highly tumorigenic human breast cancer stem cells [17] and malignant myoepithelioma breast cancer cells. [18] Even though great evidence indicated the important roles of miR-125b in the biological properties of breast cancer, few reports to date studied the relationship between miR-125b and the metastatic potential of breast cancer. The expression of miR-125b was low in most breast cancer cell lines MCF-7, T47D, SK-BR3, BT-20 and MDA-MB-175 compared to mammary epithelial cell MCF-10A, but high in MDA-MB-231 which is a highly metastatic breast cancer cells. [19] Based on the evidence above, it is theoretically proposed that the expression of miR-125b is closely related to the metastatic activity of tumor cells.

Tumor metastasis is the most prominent problem in clinical treatment of cancer, as most cancer mortality is associated with disseminated disease rather than the primary tumor. There are two leading theories about the origin of metastasis, Epithelial to mesenchymal transition (EMT) hypothesis [20], [21], [22] and cancer stem cell hypothesis. [23] EMT, characterized as loss of polarity and epithelial markers (including junctional and cell-cell adhensive proteins), [24] plays a role in cellular differentiation and tumor invasion. Cancer stem cell hypothesis suggests that cancer stem cells could initiate a primary tumor, and thus be able to initiate metastasis. It is well known that breast cancer stem cells have mesenchymal features and correlate with metastasis and poor prognosis. [25] Markers are used to define the mesenchymal phenotype such as vimentin, α-SMA (smooth muscle alpha-actin), N-cadherin and loss of E-cadherin. [21] To fully elaborate the influences of miR-125b on the mesenchymal features of breast cancer cells will provide important information on the mechanisms of tumor metastasis.

Although miR-125b has been implicated with functions as a tumor suppressor in breast tumors, its functional role in metastasis has not been clearly described. In the present study, we began by examining the function of miR-125b on cell migration. Interestingly, we found that miR-125b has a role of pro-metastasis in vitro. To further validate this pro-metastasis role, we designed a node mice model and the same result has been obtained. In addition, we investigated the mechanisms of pro-metastasis for miR-125b and found that STARD13, (StAR-related lipid transfer domain containing 13) also known as DLC2 (deleted in liver cancer cells) with a Rho-GAPase-activating protein (RhoGAP), [26] was a target protein of miR-125b. The activation of STARD13 was responsible for MCF-7 metastasis induced by miR-125b was also validated. Further examination of the consequences of gain- and loss-of-function of miR-125b in breast cancer cells, we investigated the influence of miR-125b on the expression of α-SMA and vimentin, two classical mesenchymal phenotype markers and provided new insights into the function of miR-125b in cancer metastasis.

## Materials and Methods

### Reagents, Cell Culture, and Treatment

MCF-7, MDA-MB-231, MDA-MB-435, MCF-10A, MCF-7/ADR and HEK-29 cells were obtained from the ATCC (Manassas, Virginia, USA). MCF-7 and MCF-7/ADR were cultured in RPMI 1640 medium (Gibco/BRL, Grand Island, NY) containing 10% calf serum (CS); MDA-MB-231 was cultured in L-15 medium containing 10% fetal bovine serum (FBS); MDA-MB-435, MCF-10A and HEK-293 was cultured in complete medium DMEM (Gibco) containing 10% fetal bovine serum (FBS) with 1 mM L-glutamine. All medium used in the study were supplemented with penicillin and streptomycin. miRNA over-expression and inhibition: miR-125b-mimics, inhibitors (5′-ucacaaguuagggucucaggga-3′ ) and Normal Control (NC) were designed through the reference of miRbase Database (www.miRbase.org and ) and synthesized in Biomics Biotechnology Inc (Biomics, Jiang Su, China) with OMe modification, 50 nM of which were transfected into cells using Lipofectamine 2000 (Invitrogen, Carlsbad, CA). STARD13 antibody (sc-67843) were purchased from Santa Cruz Biotechnology Inc (Santa Cruz, California, USA), vimentin antibody (catalog#2707-1) and α-SMA antibody (catalog#1184-1) form Epitomics Inc (Epitomics, California, USA).

### mRNA and miRNA Quantification

Total RNA was extracted using Trizol reagent (Invitrogen, USA). Reverse transcription-PCR was performed with M-MLV (Promega, USA ) following standard protocols.

For miRNA qPCR, the miR-125b primer, U6 primer and EzOmics SYBR qPCR kit were purchased from Biomics. Amplification procedure was as follows: 94°C for 5 min, followed by 30 cycles at 94°C for 30 s, 61°C for 45 s, finally by 72°C for 10 min. For RT-PCR, The primer sequences of various genes were demenstrated as follows: GAPDH: 5′-AAGGTCGGAGTCAACGGATT-3′, and 5′-CTGGAAGATGGTGATGGGATT-3′; STARD13: 5′-AGCCCCTGCCTCAAAGTATT-3′, and 5′-ATGGGCGTCATCTGATTCTC-3′; Vimentin: 5′-CCCTCACCTGTGAAGTGGAT-3′, and 5′-TCCAGCAGCTTCCTGTAGGT-3′; α-SMA: 5′-CATCATGCGTCTGGATCTGG-3′, and 5′-GGACAATCTCACGCTCAGCA-3′. Amplification procedure was 94°C for 5 min, followed by 30 cycles at 94°C for 30 s, 57°C for 45 s, 72°C for 45 s, finally by 72°C for 10 min.

### Adhesion Assay

Cell adhesion assay was assayed as described previously with sight modifications. [27] Microtiter wells were coated with fibronectin (Sigma, St. Louis, Missuouri) overnight. The wells were blocked for 30 min with 0.5% BSA in PBS. Cells were trypsinized and suspended at a final concentration of 5×10^5^ cells/ml in serum-free medium. miR-125b-mimics, miR-125b-inhibitor and NC were transfected into the cells for 24 h prior to seeding. The colorimetric MTT-assay was used to determine the number of remaining cells (adherent cells).

### Wound Healing Assay

MCF-7 cells were seeded into a 6-well plate and allowed to grow to 70% confluency in complete medium. Cell monolayers were wounded by a plastic tip (1 mm) that touched the plate as described previously. [28] Wounded monolayers were then washed for several times with PBS to remove cell debris and transfected with miR-125b-mimics and miR-125b-inhibitor and incubated for 24 h. Cells migrated into wound surface and the average distance of migrating cells was determined under an inverted microscopy at designated time points.

### Transwell Migration Assay

Transwell migration assays were carried out using 24-well MILLIcell Hanging Cell Culture inserts 8 µm PET(MILLIPORE) as described previously. [29] Briefly, Cells harvested 48 h after transfection using 5 mM EDTA in PBS, were added (1.25×10^5^ cells/well) in serum-free medium to triplicate wells of BD BioCoatTM MatrigelTM Invasion Chambers (BD Bioscience) and complete medium containing 10% FBS or CS was added to the lower chamber. The invasion chambers were processed for 24 h followed the manufacturer’s protocols, and migrated cells were stained using methanol and viola crystalline solution. Five random fields from each of the triplicate invasion assays were counted using phase contrast microscopy.

### Plasmid Construction and Establishment of Stable miR-125b Over-expressing Cells

The procedure of pre-microRNA plasmid construction was referred to the method described by Galardi et al. [30] In brief, the pre-miR-125b sequences were amplified by PCR from human genomic DNA using the following primers: forward/HindIII, 5′-GGC AAG CTT AAC ATT GTT G CGC TCC TCT CA-3′; reverse/BamHI, 5′-TAT GGA TCC TTC CAG GATGCA AAA GCA CGA-3′. After being digested with HindIII and BamHI, the PCR product was cloned into pSilencer 4.1 vector (Ambion, Austin, USA) and the constructs were verified by DNA sequencing. Expression of miR-125b was detected by qRT-PCR analysis after 48 h from transfection pSilencer-125b and pSilencer-control plasmid into human breast cancer cells MCF-7 for 48 h. Positive cells were selected with 2 ug/ml G418 was confirmed by qRT-PCR.

### Animal Models

Five- to six-week-old female athymic BALB/c nude mice were purchased from Hospital of Nanjing Military Region (Jiang Su, China). All experimental protocols were approved by Ethics Committee for Animal Experimentation of China Pharmaceutical University. Experimental metastasis assays: 1×10^6^ cells (for MCF-7 or MCF-7-125b) suspended in 100 µl of serum-free medium were injected into the lateral vein of six-week-old female athymic BALB/c nude mice. The NIR-imaging model was referred to the method described by Liu F and et al. [31] For NIR near infrared imaging, 3 weeks after the injection cells, each mouse was injected via tail vein with 200 µl folate-polyethylene glycol. The subjected mouse was firstly anesthetized and imaged in NIR-imaging system at predetermined intervals of 0 h and 4 h. To further confirm the pathological changes in NIR metastasis model, the mice were euthanized after injection for 24 h. The lungs, liver and kidney were dissected out, photographed, fixed in 4% PFA overnight, cry-protected in 30% sucrose in PBS and frozen in OTC embedding media (Tissue Tek) and stained with H&E.

### Luciferase miRNA Target Reporter Assay

pMiR-Report Fluc vectors (Ambion) was used to introduce the portion of the 3’ UTR of STARD13 mRNA containing the putative binding site for miR-125b. Both sense and antisense oligonucleotide templates were synthesized as 59-mer and annealed as described. The annealed oligonucleotides were digested and ligated at HindIII and SpeI sites into pMiR-Report. The sequences used in these studies were:

STARD13-wt:

5′-CTAGTTTTTGCCCAGTGTGACATCAAACTCAGGGAAGAGGAAGCTAAAGTGACGAGTGA-3′ 5′AGCTTCACTCGTCACTTTAGCTTCCTCTTCCCTGAGTTTGATGTCACACTGGGCAAAAA-3′

STARD13-mut:

5′-CTAGTTTTTGCCCAGTGTGACATCAAAACCGTAGGAGAGGAAGCTAAAGTGACGAGTGA -3 5′-AGCTTCACTCGTCACTTTAGCTTCCTCTCCTACGGTTTTGATGTCACACTGGGCAAAAA-3′

HEK 293T cells were co-transfected with the pMiR-Report vectors containing the STARD13 3’UTR with wild-type (wt) or mutant (mut) sequences and mimics-miR-125b, inhibitor or NC. The cells were lysed and luciferase activity was measured using a luminometer 48 h later. An expression cassette for Rluc was co-transfected and used to normalize the Fluc values expressed from the pMiR-Report constructs.

### Western Blotting

After various treatments for indicated intervals, cells were lysed in lysis buffer. The cells used for phospho-lysates were lysed with lysis buffer enriched with protease inhibitors cocktail, 1 mM sodium fluoride, and 1 mM sodium orthovanadate. Protein (40 µg) were separated on a 12% SDS-polyacrylamide gel and transferred electrophoretically onto polyvinylidene difluoride membranes (Millipore, USA). The membranes were blocked with 10% milk in Tris-buffered saline/0.1% Tween 20 for 2 h, subsequently blotted with primary antibodies then blotted with horseradish peroxidase-conjugated secondary antibody for 1 h. The protein bands were visualized with enhanced chemiluminescence detection system (Amersham, UK). Protein levels were quantified by density analysis using Quantity One software (BioRad).

### Trnasfection of siRNA

Short interfering RNA against the human STARD13(DLC2) sense: 5′-CCCUGCCUCAAAGUAUUCAdTdT-3′; anti-sense: 5′-UGAAUACUUUGAGGCAGGGdTdT, and the universal negative control siRNA were used. The siRNAs were transfected into MCF-7 by using Lipofectamine2000 (Invitrogen, Carlsbad, CA) per the manufacturer’s protocol.

### Immunofluorescent Assay

Cells were seeded into a 6-well plate, after various treatments, the cells at indicated intervals were fixed in 4% paraformaldehyde for 20 min and blocked with 3%BSA in PBS for 1 h at room temperature. Subsequently blotted with primary antibodies (Vimentin antibody Epitomics catalog#2707-1) and then blotted with FITC- conjugated secondary antibody for 1 h then blotted with DAPI fluorescence for 1 h and observed with the confocal laser scanning.

### Statistical Analysis

The experimental results were shown as the mean ± SEM for each group. Data were assessed by analysis of variance (ANOVA). If this analysis indicated significant differences between the group means, then each group was compared with normal control group (NC) by using the Dunnett t (2-sided) analysis. P<0.05 was considered to be statistically significant.

## Results

### miR-125b Promoted Metastasis of MCF-7 and MDA-MB-231 Cells

**Figure 1:**
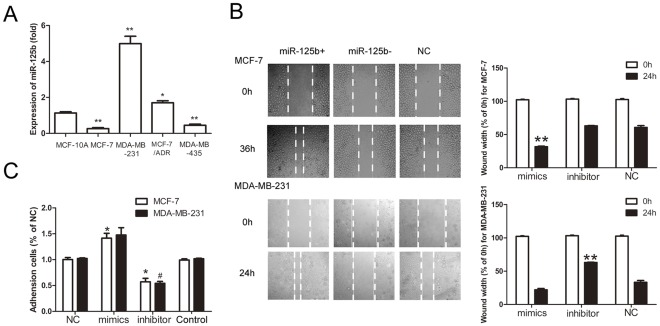
Effects of miR-125b on cell migration in MCF-7 and MDA-MB-231 in vitro. (A) Expression of miR-125b in MCF-7, MDA-MB-435, MCF-7/ADR, MDA-MB-231and MCF-10A cells measured by real-time RT-PCR. The normalized miR-125b expression for MCF-10A was set 1. Data were present as mean ± SEM, n = 3, *p<0.05, **p<0.01 vs. MCF-10A. (B) Effects of miR-125b on MCF-7 cell migration in vitro. Movement of MCF-7 and MDA-MB-231 cells into the wound was shown for mimics,inhibitor tranfected and untranfected cells and quantification of the wound healing assay. Data are presented as mean±SEM of three separate experiments, n = 3**p<0.01 vs. control group. (C) Effects of miR-125b on adhesion to fibronectin of MCF-7 and MDA-MB-231 cells. Data were present as mean ± SEM, n = 3, *p<0.05 vs. NC group in MCF-7 and #p<0.05 vs. NC group in MDA-MB-231 cell lines.

q-RT-PCR was used to test the expression of miR-125b in different metastatic potential breast cancer cell lines of MCF-7, MDA-MB-231, MCF-7/ADR MDA-MB-435, and MCF-10A. Results in Fig.1A showed that the expression of miR-125b in MDA-MB-231, highly metastatic tumor cells, was 5.35 folds more than MCF-10A and 22.6 folds than MCF-7 (Figure 1A).

Cell motility was a measure of metastatic potential of cancer cells. The motility of human breast cancer cells lines MCF-7 and MDA-MB-231 were examined by wound healing assay when treated with mimics, inhibitor and NC. Confluent monolayers of cells were scratched to be wounded and cultured for 24 h or 36 h. (Figure 1B) The treatment with miR-125b mimics led to significantly increase of wound healing cell migration compared to the treatment of NC and inhibitor of miR-125b in MCF-7 cells. For miR-125b high-expression cell lines MDA-MB-231, cell migration was inhibited when treated with miR-125b-inhibitor.

Adhension of tumor cells to extra-cellular matrix and basement membranes were considered to be the initial step in the invasive process for metastatic tumor cells. We examined the influence of miR-125b on the adhension activities of breast cancer MCF-7 and MDA-MB-231 cells to the substrates precoated with fibronectin, which is a basement member component. After transfected with mimics-miR-125b, the adhesion activity was increased about 1.42 fold in MCF-7; when treated with miR-125b inhibitors, the adhesion activity was decreased compared with NC group and the inhibition rate was about 45.8% (Figure.1C).

**Figure 2:**
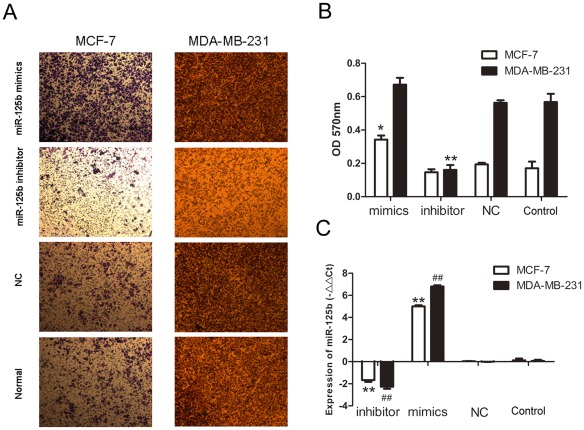
Effects of miR-125b on cell invasion in MCF-7 and MDA-MB-231 in vitro. (A) Photographs of the cell invasion through the polycarbonate membrane stain by crystal violet. The migratory cell numbers of both cell lines transfected with miR-125b mimics were significantly more than that of cells transfected with inhibitor respectively. (B) OD570 of stain crystal violet. Data were present as mean ± SEM, n = 3, *p<0.05, **p<0.01 vs. NC group. (C) Real-time PCR analysis of the expression of miR-125b with transfected miR-125b-mimics, miR-125b inhibitors, NC and untranfected one in MCF-7 and MDA-MB-231 cells. −ΔΔCt = − (Ctx-CtU6x)-(CtNCx-CtU6_NC_). Data were present as mean ± SEM, n = 3, **P<0.01 vs. NC group in MCF-7, ^##^P<0.01 vs. NC group in MDA-MB-231.

To investigate whether the differential expression of miR-125b was correlated with tumor invasion, MCF-7 and MDA-MB-231 cells were transfected with mimics-miR-125b, inhibitor-miR-125b and nonspecific control miRNA (NC) into MCF-7 and MDA-MB-231 cells, transwell migration assay was used to investigate metastasis activity. As shown from the images in Fig.2A, when the expression of miR-125b was upregulated by mimics-miR-125b in MCF-7 cell lines, the cells demonstrated high-migration potentiality compared to cells treated with inhibitor-miR-125b or NC. While the expression of miR-125b was down-regulated by adding inhibitor-miR-125b in MDA-MB-231 cell line, the cells migration ability was decreased significantly (P<0.001). Interestingly, miR-125b showed a pro-metastasis function in human breast cancer cell lines MCF-7 and MDA-MB-231. qRT-PCR was used to analyzed the expression of miR-125b when treated with mimics, inhibitor and NC and the result showed that the expression of miR-125b was positive correlation with the migration ability of breast cancer cell lines MCF-7 and MDA-MB-231 (Fig.2C).

**Figure 3:**
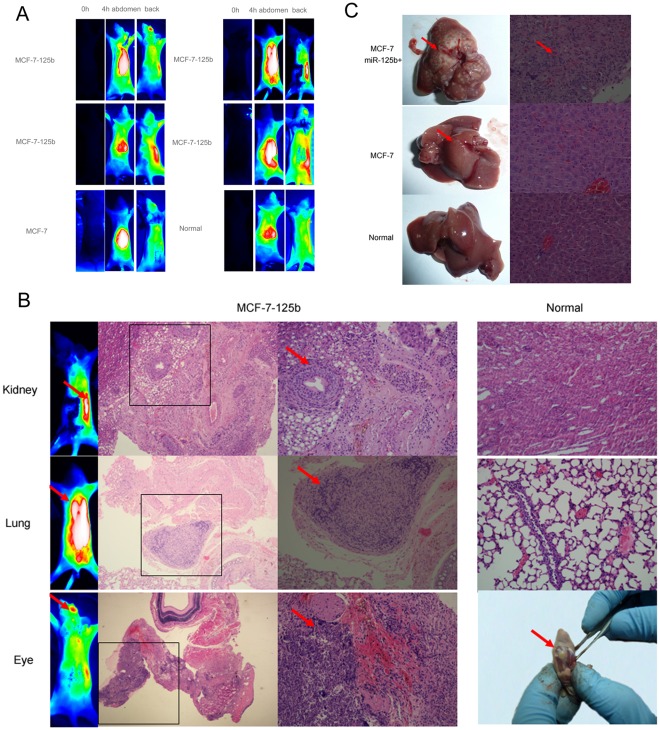
Effects of miR-125b on breast tumor metastasis in vivo. (A) Photographs of diagnosed tumor metastasis by NIR-imaging system, 3 weeks after injection breast cancer cells MCF-7 and MCF-7-125b NIR probe Folate-Polyethylene Glycol was injected to BALB/c nude mice and formation of imaged at 0 h and 4 h after injection of NIR-probe. (B) Images of metastatic focuses in patho-histological examination stained with H&E. (C) Images of representative liver dissected from mouse and patho-histological examination stained with H&E.

To test whether miR-125b was pro-metastasis in vivo, a metastasis animal model was designed. MCF-7-125b cells, MCF-7-pSilencer-control and physiological saline were injected to BALB/c nude mice and after 3 weeks injected folate-polyethylene glycol to diagnose metastatic focus. As shown in Figure 3A, in the group of up-regulated miR-125b expression, it was found that 4 cases had kidney metastasis, 1 case had lung metastasis and one had eye metastasis. To further confirm the pathological changes of NIR metastasis model, the mice were euthanized 24 h post-injection by pathologic examination. As shown in Figure 3B, we found that all of metastasis focuses developed nucleus disorder which were diagnosed by NIR metastasis model. We dissected the mouse and found one case had liver metastasis (Figure 3C) and further pathologic examination confirmed this liver metastasis arising from the metabolism of NIR probe Folate-Polyethylene Glycol was through hepato-enteric circulation.

### miR-125b Directly Targeted the Tumor Suppressor gene STARD13

**Figure 4:**
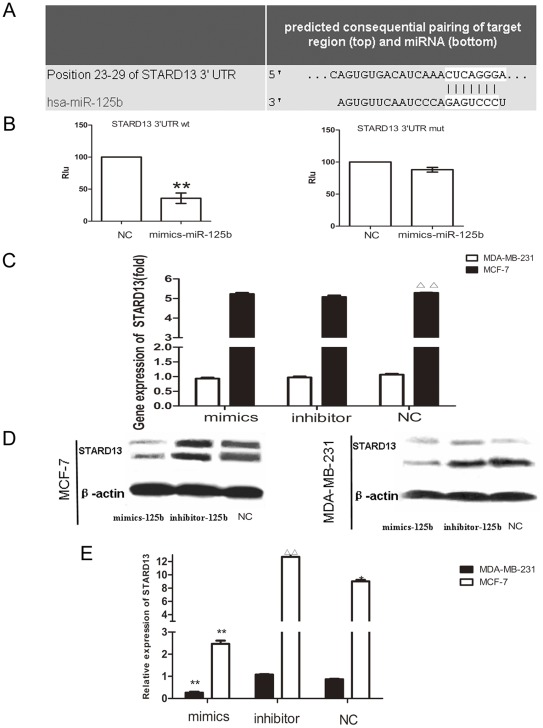
miR-125b directly target STARD13 mRNA. (A) MiR-125b directly target human STARD13. (A) The miR-125b targets on STARD13 were predicted using Targetscan 5.1. (A) pMiR-Report vectors containing the wt or mut miR-125b binding site from the 3′UTR of STARD13 mRNA, and miR-125b-mimics or NC, as well as an expression cassette for Rluc were co-transfected into HEK-293T cells. Two days later Fluc activity in cells was measured and normalized to Rluc activities two days after. Data were present as the mean ± SEM, n = 3, *p<0.05 vs. NC group. (C) Breast cancer cell lines of MCF-7 and MDA-MB-231 cells were transfected with miR-mimics, miR-125b-inhibitors and control miRNA, the gene expression of STARD13 was detected by real-time RT-PCR after 48 h. Data were present as the mean ± SEM, n = 3 ^ΔΔ^p<0.001 NC group in MDA-MB-231 vs. NC group in MCF-7; (D) Breast cancer cell lines of MCF-7 and MDA-MB-231 cells were transfected with miR-mimics, miR-125b-inhibitors and control miRNA, STARD13 protein and β-actin after 72 h were detected by western blot. (E) Quantitative data of densitometric analyses. The ratio of STARD13 protein and mRNA levels to β-actin and GAPDH were displayed as mean ± SEM, n = 3, *p<0.05, **p<0.01 vs. NC group; ^ΔΔ^p<0.001 NC group in MDA-MB-231 vs. NC group in MCF-7.

It is generally accepted that miRNAs exert their function through regulating the expression of their downstream target gene(s). To investigate the target of miR-125b in breast cancer, systemic bioinformatic publicly available algorithms were used to analyze and identify potential targets. We found that human STARD13 3′-UTR contained putative miR-125b complementary sites. A complementary site for the seed region of miR-125b was contained by STARD13 3′-UTR (Figure 4A).

To validate whether STARD13 is a bona fide target of miR-125b, a human STARD13 3’UTR fragment containing wild-type or mutant miR-125b-biding sequences was sub-cloned to the downstream of the Renilla luciferase reporter gene. When miR-125b-mimics or miR-125b-inhibitor were cotransfected with the reporter plasmid, the relative luciferase activity of the reporter containing wild-type STARD13 3’-UTR was obviously suppressed while the luciferase activity of the reporter containing mutant STARD13 3’-UTR was unaltered. It indicated that miR-125b may downregulate expression of STARD13 gene expression through miR-125b-biding sequences at the 3’UTR of STARD13 gene (Figure 4B).

To further study the relationship between miR-125b and the expression of STARD13, qRT-PCR and western bolt were performed to examine the effect of over-expression or down-regulated of miR-125b on the mRNA and protein levels of STARD13 in cancer cells. It showed that there were significant inverse correlations between the expression of miR-125b and STARD13 protein (Figure 4D and E,) for breast cancer cells. However, there was no correlation between miR-125b and the STARD13 expression in mRNA level (Figure 4C).

### miR-125b Promoted Metastasis by Targeting STARD13 in MCF-7

**Figure 5:**
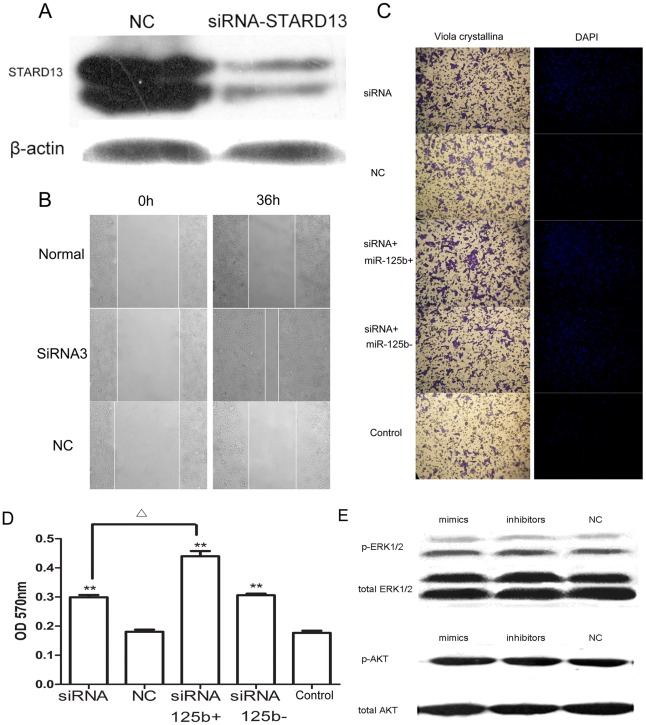
miR-125b regulated breast cancer metastasis by targeting STARD13. (A) Expression of STARD13 and β-actin were detected by western blot treated with siRNA-STARD13 and NC. (B) Effects of STARD13 on MCF-7 cell migration *in vitro*. Wound healing assay for MCF-7 cells was carried out by adding siRNA-STARD13 and NC and photographed at 0 h and 36 h. (C) Effects of miR-125b and STARD13 on cell invasion in MCF-7 cells *in vitro*. Photographs of the cell invasion through the polycarbonate membrane stained with crystal violet and DAPI. (D) OD570 of stain crystal violet. Data were present as mean ± SEM, n = 3, *p<0.05, **p<0.01 vs NC group; ^Δ^p<0.05, group of cotransfected with siRNA-STARD13 and miR-125b mimics vs group of treatment with siRNA-STARD13. (E) Effects of miR-125b on ERK1/2 and AKT phosphorylation. MCF-7 cells were transfected with miR-125b- mimics, miR-125b-inhibitors and control RNA. After 48 h, the phosphorylation of ERK1/2 and AKT was detected by western blot.

We validated that miR-125b promoted breast cancer metastasis in vivo and in vitro and that STARD13 was one of miR-125b targets was also validated in previous results. To further explore whether miR-125b promoted metastasis by targeting STARD13, a siRNA targeting STARD13 was designed. It was shown in Figure 5A, we got an expected inhibition effect after transfected with siRNA-STARD13 for 72 h. In addition, the motility of human breast cancer cells lines MCF-7 was examined by wound healing assay (Figure 5B). Confluent monolayers of cells were scratched to be wounded and cultured for 36 h. The treatment with siRNA-STARD13 led to significantly increased wound healing cell migration compared to the treatment of NC or Normal group in MCF-7 cells.

Further we investigated the relationship between STARD13 and invasion potential in breast cancer cells by transwell migration assay with co-transfection of siRNA-STARD13, miR-125b mimics and siRNA-STARD13, miR-125b inhibitor and siRNA-STARD13 and NC group. It was shown in Figure 5C, cells showed high-migration potentiality compared to cells of NC or Normal group when down-regulated STARD13 by siRNA-STARD13 (Figure 5D); the group which cotransfected with siRNA-STARD13 and miR-125b mimics displayed significantly difference compared with siRNA-STARD13 group, while the siRNA+miR-125b- group had no significant difference compared to siRNA-STARD13 group. Interestingly, these results suggested that miR-125b regulated metastasis not only by targeting STARD13 but also by some other unknown mechanisms.

Earlier Scott et al. restored that miR-125b expression in SKBR3 cells which over-express ERBB2 [32]and reported a decreased cell migration due to targeting of ERBB2 by miR-125b. We investigated the influence of miR-125b on HER2 signaling pathway in ERBB2-negative breast cancer cell lines MCF-7. [33] Western blotting for phosphorylated ERK1/2 and AKT, downstream targets of ERBB2, demonstrated that there was no significant decrease in MCF-7(Figure 5E). These results indicated that HER2 signaling pathway did not play an important role in miR-125b regulated ERBB2-negative breast cancer metastasis.

### Vimentin and α-SMA were Involved in miR-125b Regulated Metastasis

**Figure 6:**
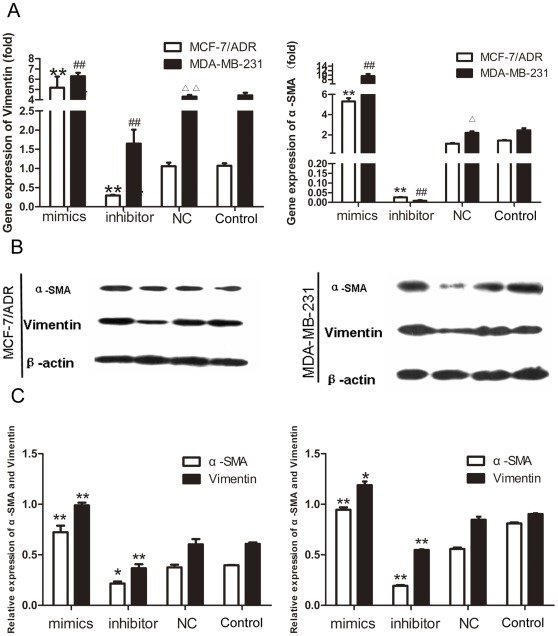
miR-125b affected vimentin and α-SMA at gene level and protein level. (A) MCF-7/ADR and MDA-MB-231 cells were transfected with miR-125b-mimics, miR-125b-inhibitors and control RNA. After 48 h, the expression of vimentin andα-SMA was detected by real-time RT-PCR. Data were present as mean ± SEM, n = 3, *P<0.05,**P<0.01 vs. NC group in MCF-7/ADR; ^#^P<0.05,^##^P<0.01 vs. NC group in MDA-MB-231; ^ΔΔ^P<0.01 NC group in MDA-MB-231 vs. NC group in MCF-7/ADR. (B) Expression of vimentin and α-SMA by western blot with got or lost functions of miR-125b. (C) Representative quantitative data of densitometric analyses. The ratios of vimentin and α-SMA to β-actin were present as mean ± SEM, n = 3, *p<0.05, **p<0.01 vs. NC group.

Epithelial to mesenchymal transition (EMT), characterized as loss of polarity and epithelial markers (including junctional and cell-cell adhesion proteins), has long been known to play a role in cellular metastasis and tumor invasion. Markers used to define the mesenchymal phenotype include the upregulation of vimentin, α-SMA and N-cadherin, and loss of E-cadherin. To further examine whether miR-125b would induce EMT, breast cancer cell lines MDA-MB-231 and MCF-7/ADR were used to observe the variation of vimentin and α-SMA because MCF-7 is vimentin-negative cell line. Consistent with metastasis ability, after transient transfection with miR-125b-mimics in MCF-7/ADR, both vimentin and α-SMA mRNA levels were increased by 5.2 and 6.34 fold compared to NC group (Figure 6A). Protein levels of vimentin and α-SMA were also increased (Figure 6B) by 2.70 and 1.92 fold (Figure 6C). In contrast, mRNA and protein levels of Vimentin and α-SMA were reduced by 97.4%, 98.15% and 64.72, 34.66% in MDA-MB-231 with miR-125b-inhibitor transient transfection.In sum, the results indicated that mRNA and protein levels of vimentin and α-SMA were positively correlated with the expression of miR-125b. It suggested that miR-125b was important in regulating reorganization of actin cytoskeleton and the maintenance of cell morphology.

**Figure 7:**
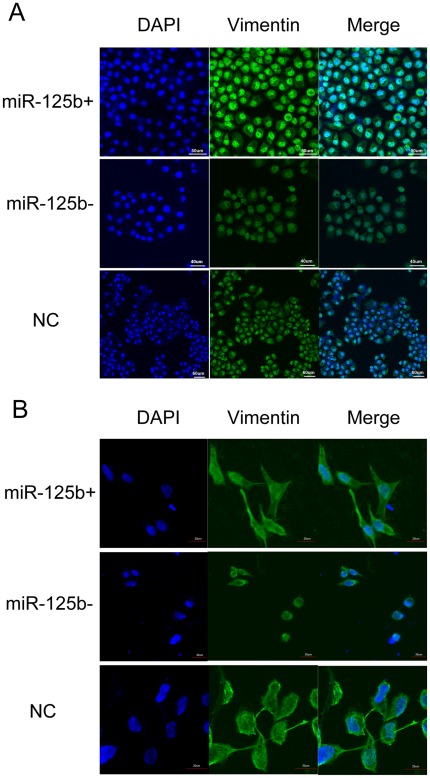
Immunofluorescence analysis of vimentin expression. miR-125b-mimics, inhibitor and NC were transfected into MDA-MB-231 and MCF-7/ADR, respectively. Green, vimentin was immunostained with anti-vimentin; blue, nuclei were stained with DAPI.

Immunofluorescent assay was used to investigate the effect of miR-125b on expression and distribution of vimentin in breast cancer cell lines MDA-MB-231 and MCF-7/ADR. The expression of vimentin was up-regulated in MCF-7/ADR cells when treated with miR-125b-mimics; (Figure 7A) while the expression was down-regulated compared with NC or normal control group in MDA-MB-231 cells (Figure 7B). Meanwhile, the morphology of cells treated with miR-125b-inhibitor changed from long shuttle-shape to spherical.

### miR-125b Regulated α-SMA by STARD13-RhoA-ROCK Signaling Pathway

**Figure 8:**
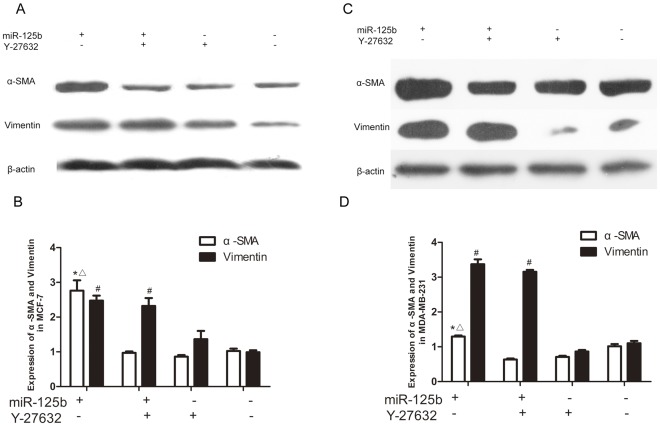
Effects of miR-125b on vimentin and α-SMA expression in MCF-7/ADR and MDA-MB-231 with ROCK inhibitor *in vitro*. (A B) Effects of miR-125b and Y-27632 on α-SMA and Vimentin expression. MCF-7/ADR and MDA-MB-231 cells were transfected with miR-125bmimics or control RNA respectively. After 24 h, cells were incubated with or without Y-27632; after 72 h, α-SMA protein, vimentin and β-actin were detected by western blot. (C D) Quantitative data of densitometric analyses. The ratio of α-SMA protein to β-actin were present as mean ± SEM, n = 3, *p<0.05, vs. NC group of α-SMA expression ^#^p<0.05, vs. group of cotransfected with miR-125b and Y-27632 of α-SMA expression.

STARD13 (DLC2), a tumor suppressor protein, has growth-suppressive and anti-metastatic effects on breast cancer cell lines MCF-7/ADR and can inhibit the activity of RhoA. [34] We found that miR-125b targeted STARD13 and miR-125b might play a role in regulating vimentin and α-SMA expression. Therefore, we speculated that inhibition of RhoA-ROCK in MCF-7/ADR might affect the regulation function of miR-125b on vimentin and α-SMA expression. To confirm these ideas we used an inhibitor of ROCK to study the expression of vimentin and α-SMA. When MCF-7/ADR and MDA-MB-231 cells were treated with Y-27632 (inhibitor of ROCK) after transient transfection with miR-125b-mimics, it completely blocked the upregulation of α-SMA induced by miR-125b rather than the regulation of vimentin (Figure 8). It suggested that miR-125b regulated α-SMA in MCF-7 cells through STARD13-RhoA-ROCK signaling pathway.

## Discussion

MicroRNAs are known to regulate the expression of genes involved in the control of tumor development, proliferation, apoptosis and stress response. [35], [36], [37], [38] It has been demonstrated that miRNAs aberrantly expressed in many human cancers, including breast cancers, but their functions and mechanisms in tumorigenesis isn’t explicit. Our results obtained from gain-of-function and loss-of-function approaches indicated that miR-125b was positively correlated with the invasive and migratory abilities of breast cancer cells in vitro. For human breast cancer cell lines MCF-7 with low metastatic ability and low expression of miR-125b, up-regulation of the expression of miR-125b permitted MCF-7 cell lines to gain a high metastasis potentiality (Figure1B 2A and Figure 3). In MDA-MB-231 cell lines with high expression of miR-125b, the cells migration ability was decreased significantly with down-regulation of the expression of miR-125b by the miR-125b inhibitor (Figure2A). In the study of nude mice model, we validated that miR-125b had a role of pro-metastasis *in vivo* by a probe for NIR-imaging. (Figure 3) Using tumor targeting probe could to determine metastasis focuses widely, conveniently, accurately compared to traditional metastasis experiment.

miRNAs regulate functions of their target genes by binding to the complementary regions of messenger transcripts to repress their translation or to regulate their degradation. STARD13 encodes a protein that contains a sterile alpha motif domain in the N-terminus, an ATP/GTP-binding motif, a GTPase-activating protein domain, and a STAR-related lipid transfer domain in the C-terminus [26], [39] STARD13, a putative tumor suppressor gene located at chromosome 13q12.3 that has loss of heterozygosity in hepatic cancer. Recombinant DLC2 showed GAP activity which is specific for small GTPases RhoA and CDC42 (cell division cycle 42). STARD13 is lower expressed in breast cancer cell lines compared with normal cells. It suppresses cell growth and migration via the regulation of Raf-1-ERK1/2-p70S6K signaling pathway in Hepatocellular carcinoma cells [34], [40]. For the first time, we identified STARD13 as a target of miR-125b (Figure 4) and miR-125b would promote breast cancer cells migration via regulating STARD13 expression (Figure 5B and 5C) by a siRNA targeting STARD13. Interestingly, our results(Figure 5C and 8)showed that MCF-7 cells got a higher metastasis potentiality compared to siRNA group when co-transfected with miR-125b-mimics and siRNA-STARD13. It may suggest that miR-125b regulate metastasis not only through targeting STARD13 but through also by some other unknown mechanisms.

The question of whether miR-125b is a proto-oncogene or anti-oncogene in breast cancers is controversial. Scott et al. reported that oncogene ERBB2 was a target of miR-125b [32] but in another report, Zhou et al. found that miR-125b down-regulated the expression of pro-apoptotic BCL-2 antagonist killer1 (Bak1) and miR-125b have no influence on expression of ERBB2 in breast cell lines BT-474, BT-474M1 and SKBr3. [41] For a long time, miR-125b was considered as an anti-oncogene because of miR-125b was down-regulated in breast cancer. But lasted research shown that miR-125b was a basal-like microRNAs [18] and was significantly elevated in highly tumorigenic human breast cancer stem cells. [17] Patients with triple-negative tumors have a relatively poor outcome and cannot be treated with endocrine therapy or therapies targeted to human epidermal growth factor receptor type 2 (HER2). [42] Most basal-like breast cancers was characterized by an absence or low levels of expression of ER, an absence of HER2 and often display gene-expression patterns that are consistent with those of cells undergoing EMT. In this study, a luminal subtype HER2-negative breast cancer cell lines MCF-7 [33]was used to investigate the influence of miR-125b on HER2 signal pathway. Our results shown that miR-125b has no significant to decrease phosphorylation of ERK1/2 and AKT which downstream targets of ERBB2 in HER2-negative breast cancer cell lines MCF-7 (Fig.5E).

Cell migration occurs in the course of development of different tissues and in several diseases including cancer and fibrosis by inducing the expression of α-SMA and vimentin. Our results demonstrated that miR-125b was a novel regulator for α-SMA and vimentin in breast cancer cells (Fig.6). We found that miR-125b significantly upregulated vimentin and α-SMA expression while another EMT marker E-Cadherin had no significantly changed (data not shown). Elevating vimentin and α-SMA expression lead to a high metastasis potentiality and some mesenchymal cell characteristics in breast cancer cells (Fig.6). TGF-β1-induced EMT and apoptosis are closely related to the cell cycle stage: apoptosis is induced mostly in cells at G2/M phase, whereas EMT is only induced in cells at G1/S phase. [43] miR-125b regulated G1/S transition through E2F3-Cyclin A2 signaling pathway and kept cells at G1/S phase [10] may indicate an important role of miR-125b in TGFβ1-indunced EMT. Furthermore, by knockdown of miR-125b in MDA-MB-231, expression of vimentin and α-SMA was downregulated by knockdown of miR-125b in MDA-MB-231 (Figure.6 and 7), the cell morphology was changed from long shuttle-shape to spherical and the cell migration was significantly inhibited (Figure 7B and 2A). It indicated that miR-125b was a key molecule in adjusting reorganization of actin cytoskeleton. Elevated expression of miR-125b induces luminal-like breast cancer cells to obtain post-EMT or basal-like properties, which has an aggressive phenotype characterized by high cell migration and poor clinical outcome. [44] Semblable results were reported in latest research that the content of miR-125b was significantly increased in basal-like breast cells compared to luminal cells. [18] Taken together, miR-125b plays an important role in keeping basal-like and post-EMT properties.

Expression of the STARD13 GAP domain inhibited Rho-mediated formation of actin stress fibers and suppressed Ras signaling and Ras-induced cellular transformation in a GAP-dependent manner. [45] Introduction of human STARD13 into mouse fibroblasts showed the establishment of cell–cell contacts and of cell–matrix interactions was crucial to obtain a fully polarized epithelial state. [46] Rho GTPase is required for both the establishment of a fully polarized state and a motile phenotype upon EMT. [47], [48] it can produce several factors which may stimulate proliferation of cancer cells and facilitate their infiltration. The role of miR-125b in adjusting the ultrastructure and cytoskeleton protein expression of breast cancer cell lines MDA-MB-231 is possibly through attenuating the phosphorylation of Rho-GTPases by silencing STARD13, as our results showed that miR-125b promoted metastasis by downstream signaling transduction pathways of STARD13. Rho family GTPases are greatly over-expressed in breast tumors and RhoA is necessary for Ras-mediated transformation and metastatic spread. [49], [50] To further demonstrate the mechanisms that miR-125b regulated vimentin and α-SMA, we used an inhibitor of ROCK to investigate RhoA-ROCK signal pathway. We identified that miR-125b regulated α-SMA expression through STARD13-RhoA-ROCK signaling pathway (Fig.8). miR-125b may induce the phosphorylation of Rho-GTPases ROCK by silencing STARD13, while the mechanisms of miR-125b regulating vimentin expression need to be further studied.

### Conclusions

In this study, we found that miR-125b played a critical role in breast cancer metastasis. The expression of miR-125b affected the metastatic activities of breast cancer cells in vivo and in vitro. In addition, we found that tumor suppressor gene STARD13 was a target protein of miR-125b. Over-expression of miR-125b induced breast cancer cells to obtain epithelial and mesenchymal characteristics while regulating the reorganization of actin cytoskeleton. miR-125b up-regulated the expression of vimentin and α-SMA both at mRNA and protein levels. The regulation of α-SMA by miR-125b was dependent on STARD13-RhoA-ROCK signaling pathway. Therefore, our results supported the hypothesis that miR-125b is critical in breast cancer cells metastasis.
